# Supplementary material for: Long-read sequencing transcriptome quantification with lr-kallisto
Source: PLoS Comput Biol. 2025 Dec 1;21(12):e1013692. doi: 10.1371/journal.pcbi.1013692 (PMC12680354; doi:10.1371/journal.pcbi.1013692)
Supplement: S1 Text — Motivation: Comparison of kallisto vs lr-kallisto on PacBio 1.4% error simulation. Fig Bi. Comparison of the percent of reads mapping as spliced vs. unspliced with and without exome capture. Fig Bii. Quantifications of C57BL/6J exome capture samples using Bambu, IsoQuant, and Oarfish. Fig Biii. Runtime performance comparisons for lr-kallisto, IsoQuant, Bambu, and Oarfish. Fig Biv. I. (i) Venn diagram of barcodes in ONT and Illumina. (ii) Number of ONT UMI/nucleus vs. Spearman correlation between ONT and Illumina single-nucleus gene-level counts.II. (i) Venn diagram of barcodes in Illumina random oligo and Illumina poly dT. (ii) Number of random oligo UMI/nucleus vs. Spearman correlation between Illumina priming methods.III. (i) Venn diagram of barcodes in ONT random oligo and ONT poly dT. (ii) Number of random oligo UMI/nucleus vs. Spearman correlation between ONT priming methods. Fig Bv. Contrast of non-exome vs. exome capture in Illumina and ONT datasets. Fig Bvi. Contrast of priming methods in exome capture comparing Illumina vs. ONT data. Fig Ci. Performance of lr-kallisto on ONT-sequenced direct cDNA libraries from HCT116 cell line using Oarfish v0.3.1 (A) and v0.5.1 (B). Fig Cii. Comparison of lr-kallisto on ONT-sequenced HCT116 libraries using directRNA and direct cDNA, comparing replicates. Fig Ciii. Evaluation of Bambu, IsoQuant, lr-kallisto, and Oarfish on mouse cortex high-depth PacBio data. Created with https://BioRender.com Fig D. Evaluation of lr-kallisto, Bambu, IsoQuant, and Oarfish using LRGASP Challenge 2 metrics in Mouse ES cells. Fig E. (a) Benchmarks of Bambu, IsoQuant, lr-kallisto, and Oarfish across simulation error rates.(b) Performance on all annotated transcripts at ONT 11.2% error.(c) Performance at ONT 15.2% error. Fig F. Transcript de Bruijn Graph Bandage plots from lr-kallisto. Table A. Comparison of tools on memory usage, runtime, percent of aligned and uniquely aligned reads, and total read counts across multiple long-read quantificat [file pcbi.1013692.s001.pdf]

Supplementary Figures and Table
Long-read sequencing transcriptome quantification with lr-kallisto

Rebekah K. Loving[1], **Delaney K. Sullivan**[1,2], **Fairlie Reese**[3,4], **Elisabeth Rebboah**[3,4], **Jasmine Sakr**[3,4], **Narges Rezaie**[3,4], **Heidi Y. Liang**[3,4], **Ghassan Filimban**[3,4], **Shimako Kawauchi**[3], **A. Sina Booeshaghi**[5], **Páll Melsted**[6,7], **Conrad Oakes**[1], **Diane Trout**[1], **Brian A. Williams**[1], **Grant R. MacGregor**[3], **Barbara J. Wold**[1], **Ali Mortazavi**[3,4], **and Lior Pachter**[1,8]

[1]Division of Biology and Biological Engineering, California Institute of Technology, Pasadena, California, USA
[2]UCLA-Caltech Medical Scientist Training Program, David Geffen School of Medicine, University of California, Los Angeles, Los Angeles, California, USA
[3]Developmental and Cell Biology, University of California Irvine, Irvine, California, USA
[4]Center for Complex Biological Systems, University of California Irvine, Irvine, California, USA
[5]Department of Bioengineering, University of California, Berkeley, Berkeley, California, USA
[6]deCODE Genetics/Amgen Inc., Sturlugata Reykjavík, Iceland
[7] Faculty of Industrial Engineering, Mechanical Engineering and Computer Science, School of Engineering and Natural Sciences, University of Iceland, Sæmundargata Reykjavík, Iceland
[8]Department of Computing and Mathematical Sciences, California Institute of Technology, Pasadena, California, USA

Correspondence: ***Ali Mortazavi (ali.mortazavi@uci.edu) and Lior Pachter (lpachter@caltech.edu)***

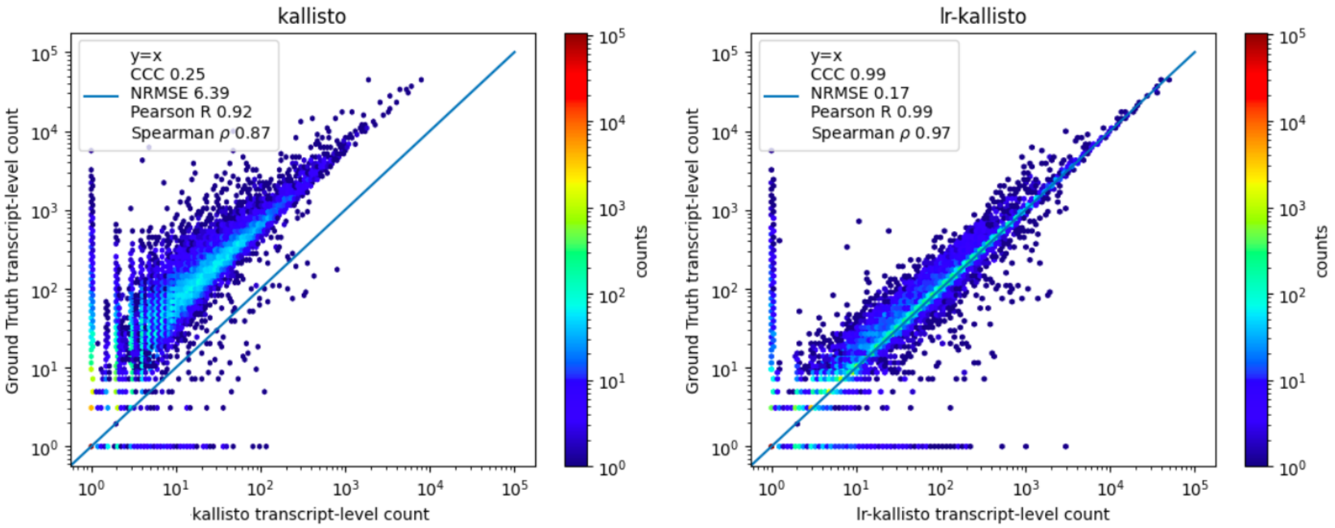

Fig A Motivation: Comparison of kallisto vs lr-kallisto on PacBio 1.4% error simulation.

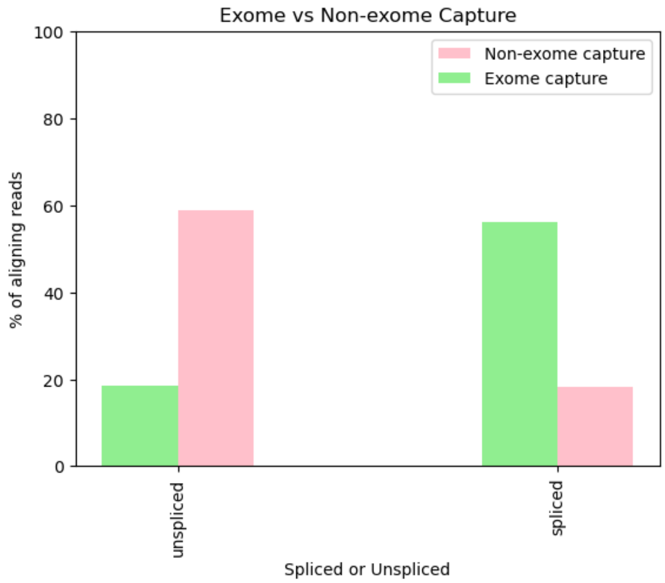

Fig Bi: Comparison of the percent of reads mapping as spliced vs. unspliced reads with and without exome capture.

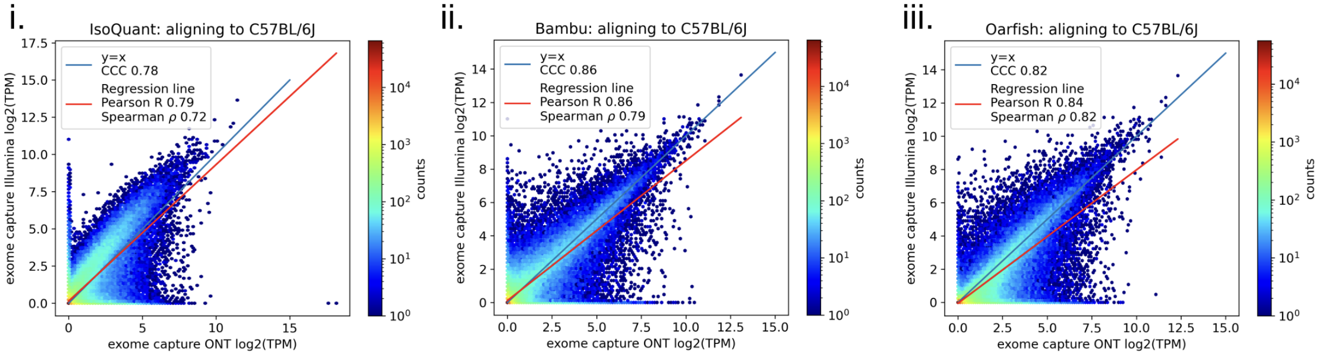

Fig Bii: Quantifications of the C57BL/6J exome capture samples with Bambu, IsoQuant, and Oarfish.

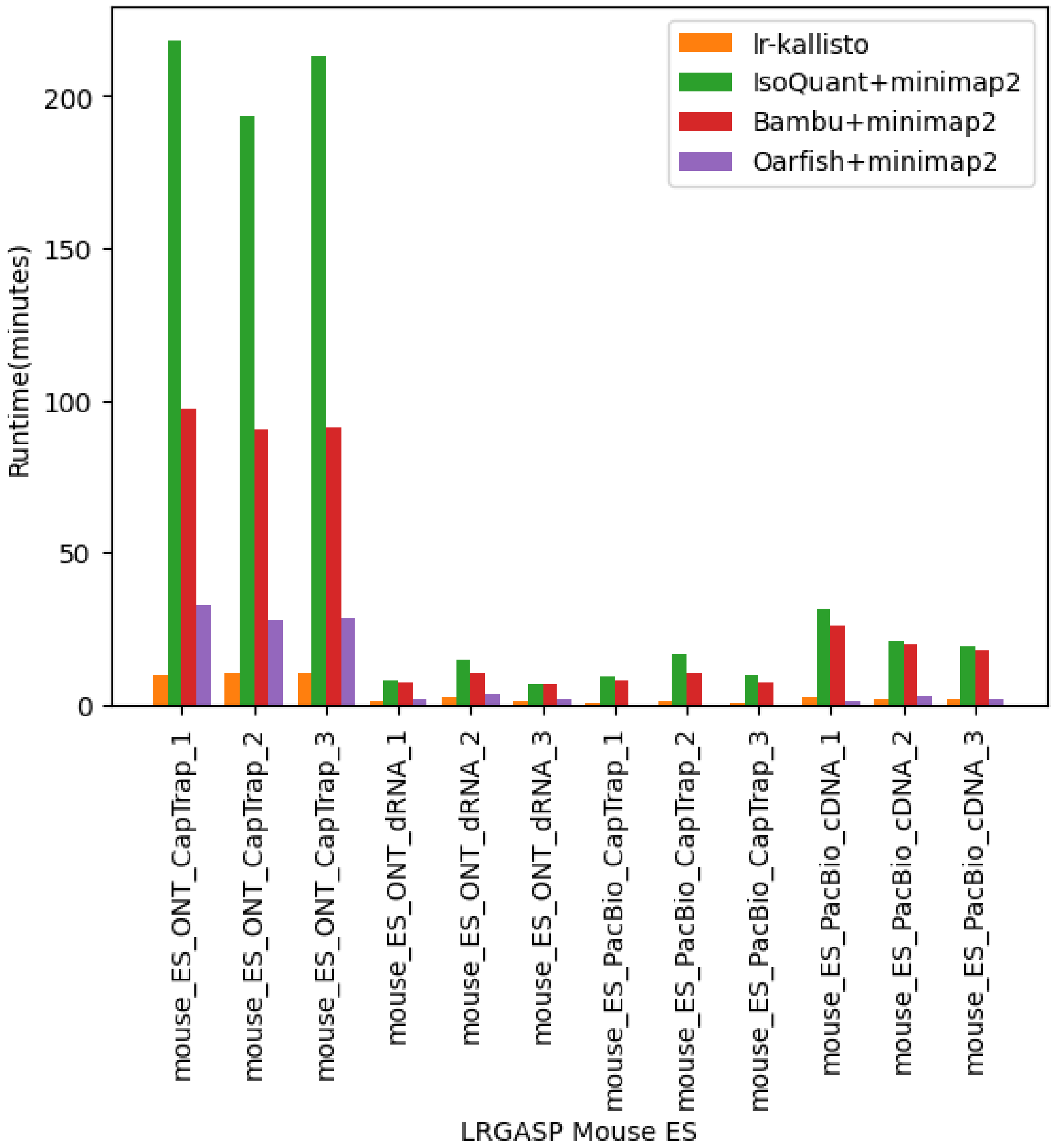

Fig Biii: Runtime performance comparisons for lr-kallisto, IsoQuant, Bambu and Oarfish.

Tool	Max RSS	hh:mm:ss	% Aligned	% Unique	Total # of Reads
lr-kallisto (d-list, pseudobam)	1.8 Gb	1:30:19	56.0%	19.6%	105,591,654
lr-kallisto (no d-list, pseudobam)	5.3 Gb	2:50:46	75.7%	28.4%	105,591,654
lr-kallisto quant-only (d-list)	1.8 Gb	0:02:03	—	—	105,591,654
lr-kallisto quant-only (no d-list)	5.3 Gb	0:01:47	—	—	105,591,654
IsoQuant (post minimap2)	≥84.3 Gb	8:59:12	—	—	105,591,654
bambu (post minimap2)	95.1 Gb	3:37:11	—	—	105,591,654
minimap2+oarfish	17.9 Gb	5:09:36	—	—	105,591,654
minimap2 (transcriptome)	5.3 Gb	4:29:15	90.3%	17.1%	105,591,654
oarfish (post minimap2)	17.9 Gb	0:40:21	—	—	105,591,654
kallisto (Illumina)	—	—	64.2%	15.7%	158,034,313
kallisto (Illumina, nac)	—	—	92.4%	29.1%	158,034,313

Table 1. Table A: Comparison of tools on memory usage, runtime, alignment rate, and uniquely aligned reads.

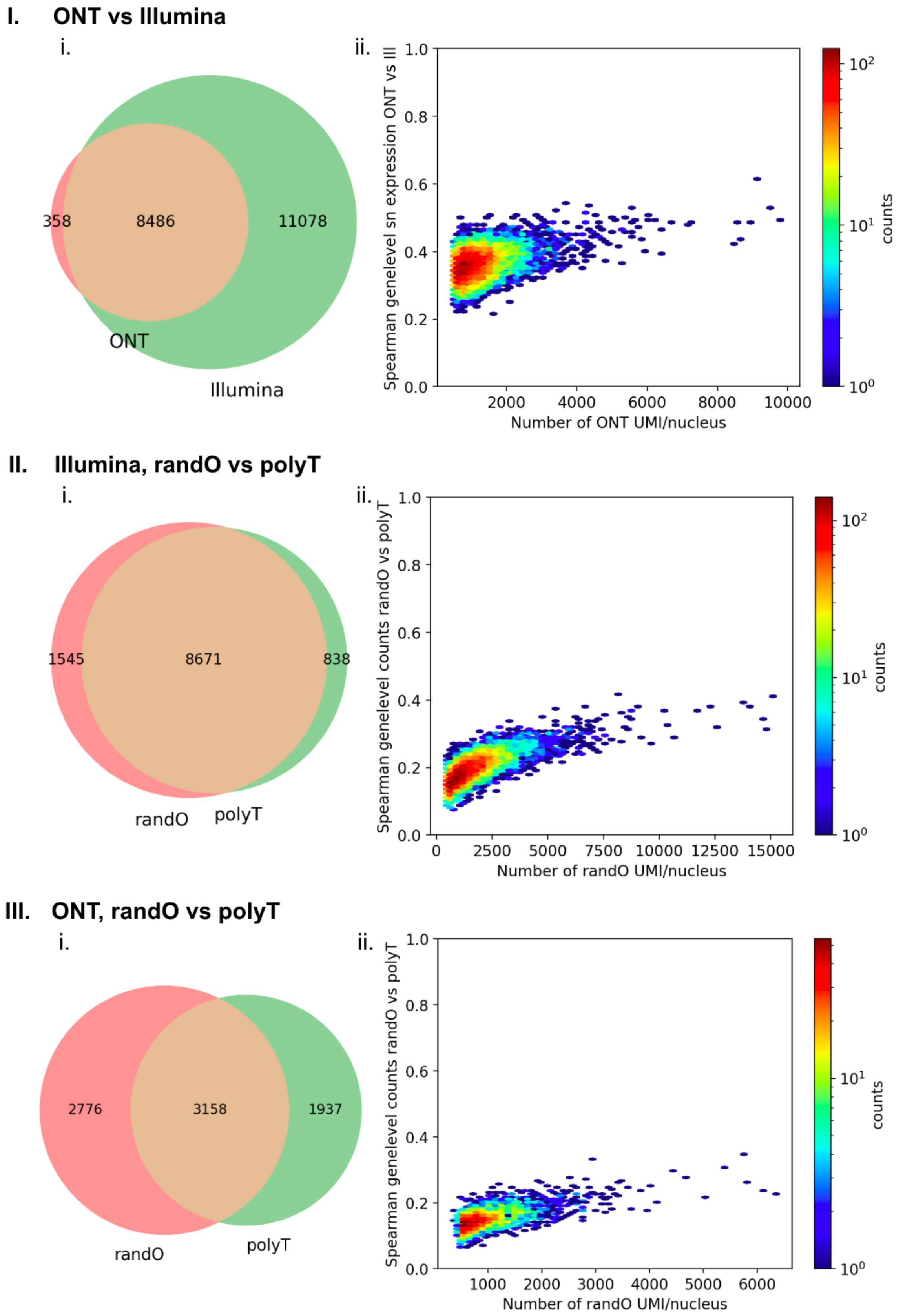

Fig Biv: I. i. Venn diagram of barcodes in ONT and Illumina. ii. Number of ONT UMI/nucleus vs Spearman correlation between ONT and Illumina single-nucleus gene level counts. II. i. Venn diagram of barcodes in Illumina random oligo (randO) and Illumina poly dT. ii. Number of randO UMI/nucleus vs Spearman correlation between Illumina randO and Illumina polydT single-nucleus gene level counts. III. i. Venn diagram of barcodes in ONT random oligo (randO) and ONT poly dT. ii. Number of randO UMI/nucleus vs Spearman correlation between ONT randO and ONT polydT single-nucleus gene level counts.

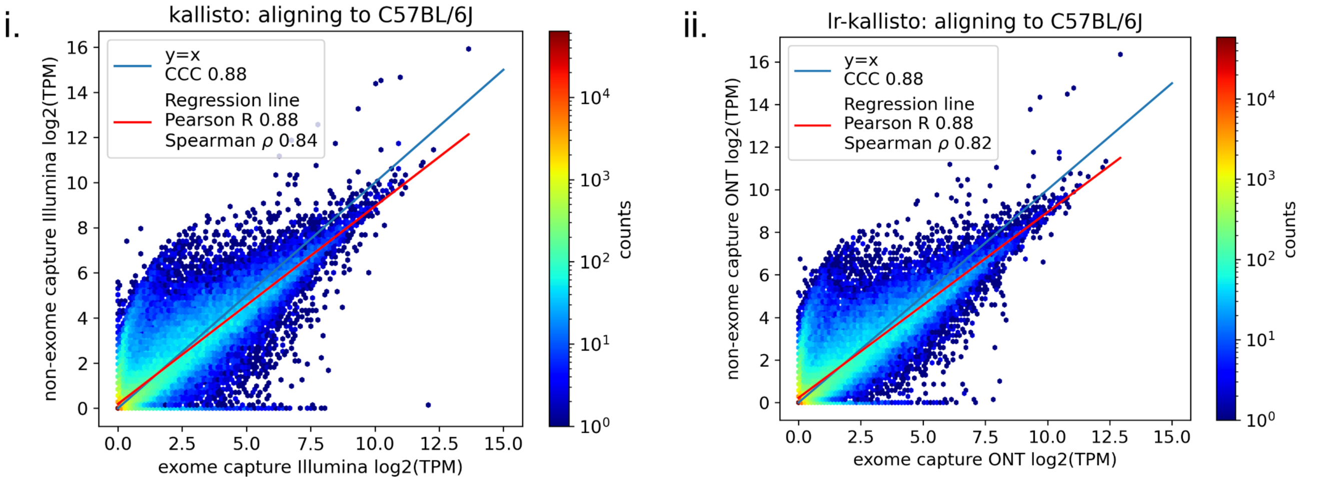

Fig Bv: Contrast of non-exome vs exome capture in Illumina and ONT.

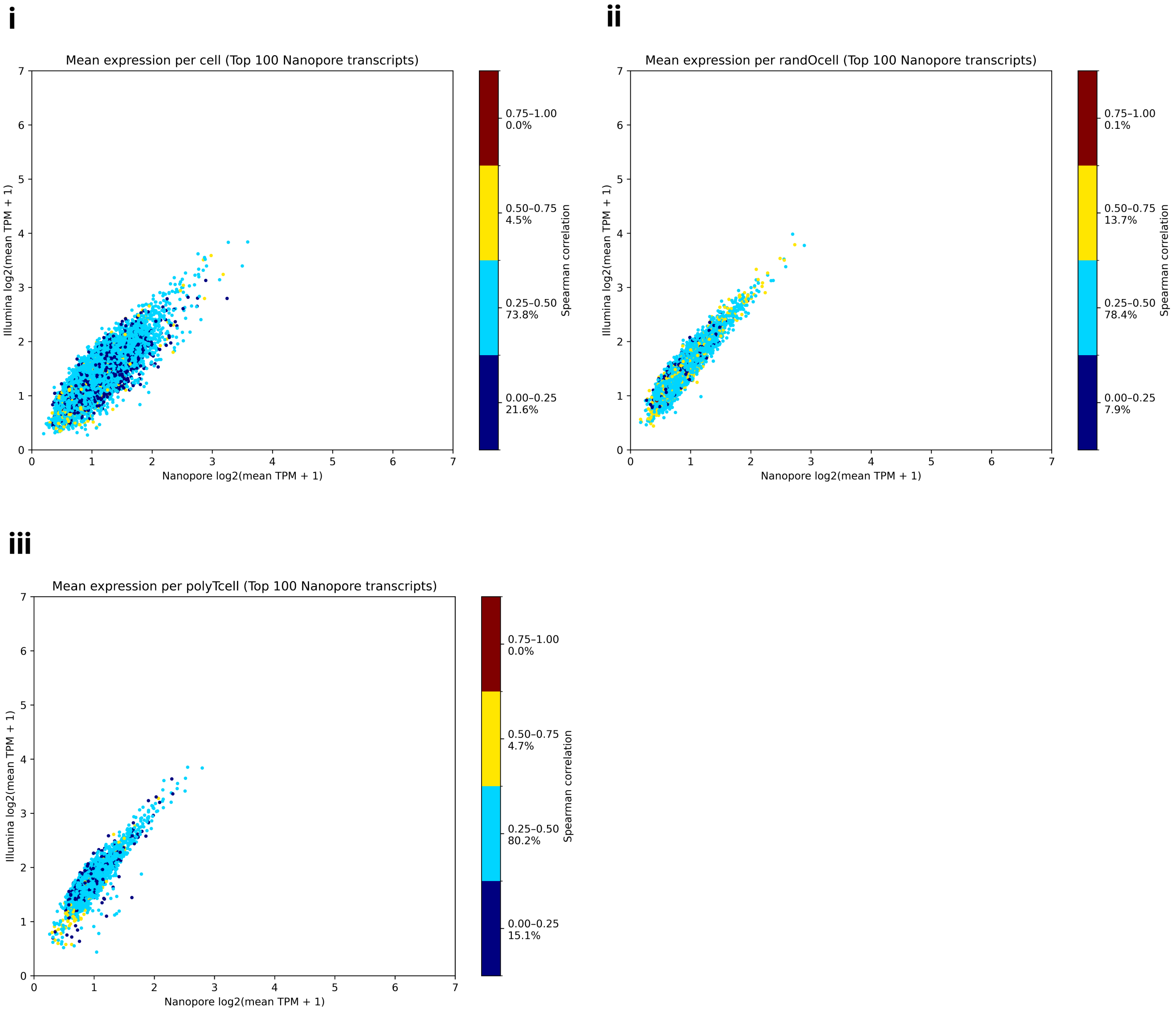

Fig Bvi: Contrast of priming methods in exome capture in Illumina vs. ONT.

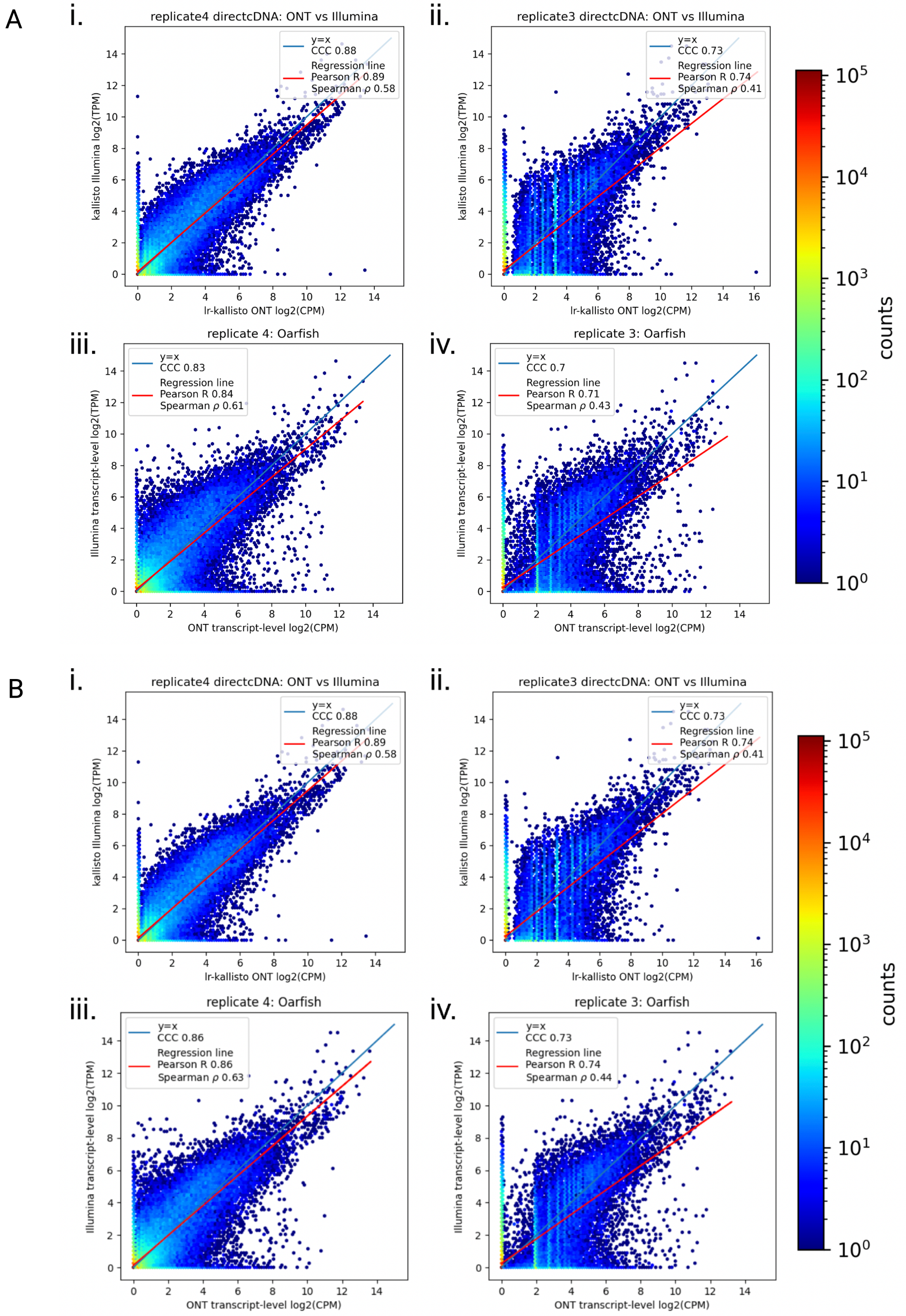

Fig Ci: Performance of lr-kallisto on ONT sequenced direct cDNA libraries from the HCT116 cell line, where panel A is with Oarfish v0.3.1 and panel B is with Oarfish v0.5.1.

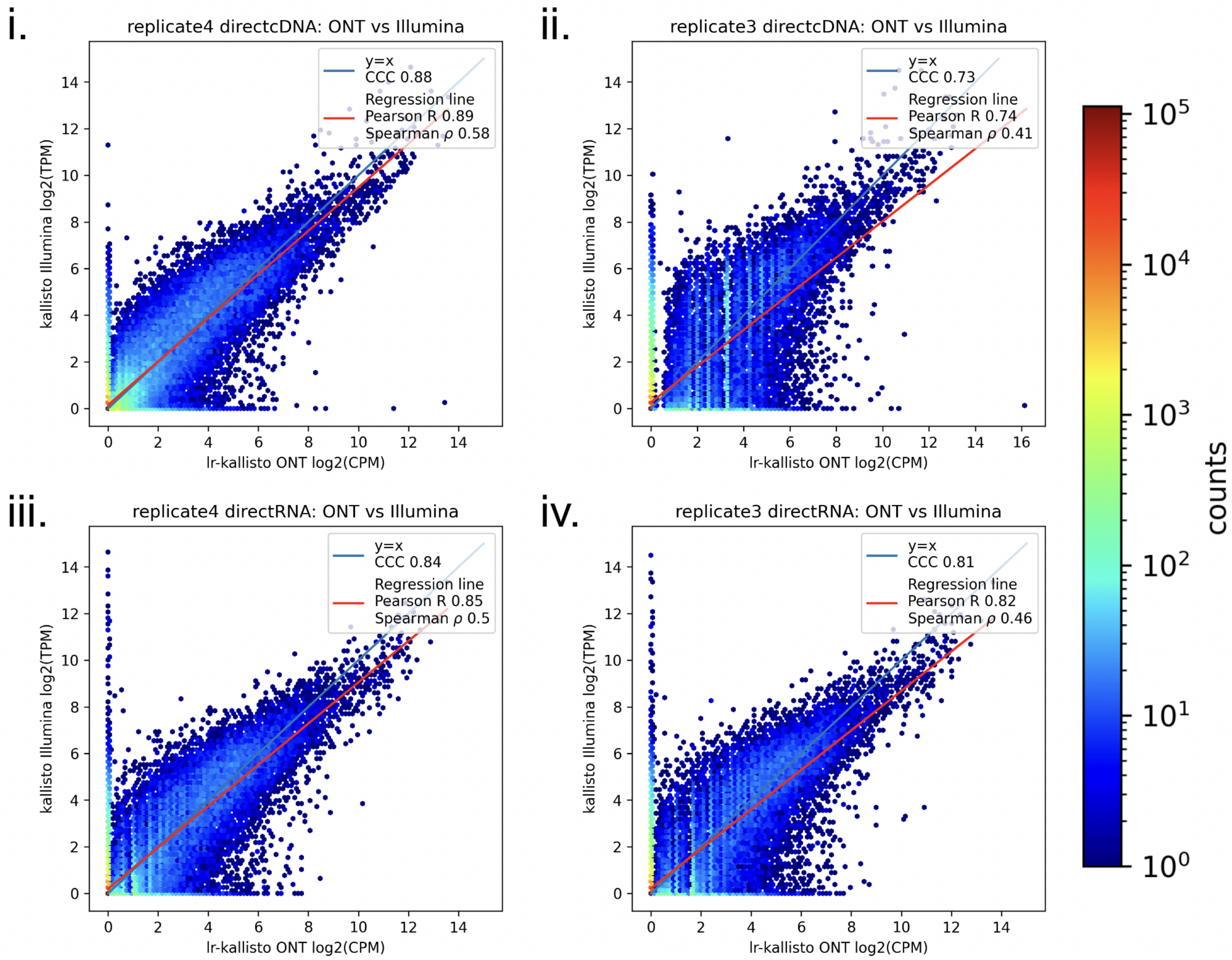

Fig Cii: Comparison of lr-kallisto on ONT sequenced HCT116 cell line libraries generated with directRNA and direct cDNA between two replicates in each.

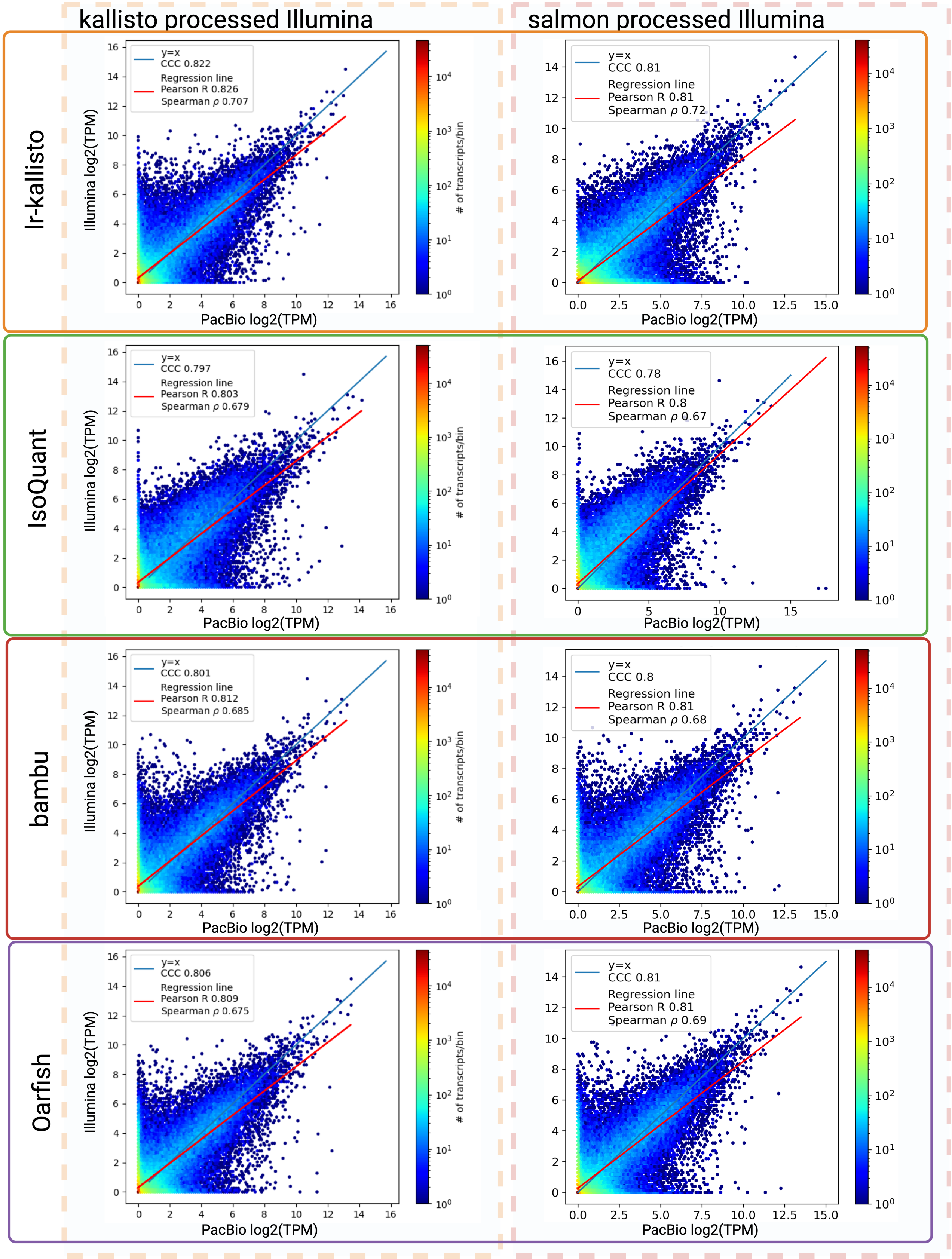

Fig Ciii: Evaluation of Bambu, IsoQuant, lr-kallisto, and Oarfish on mouse cortex high-depth PacBio data.

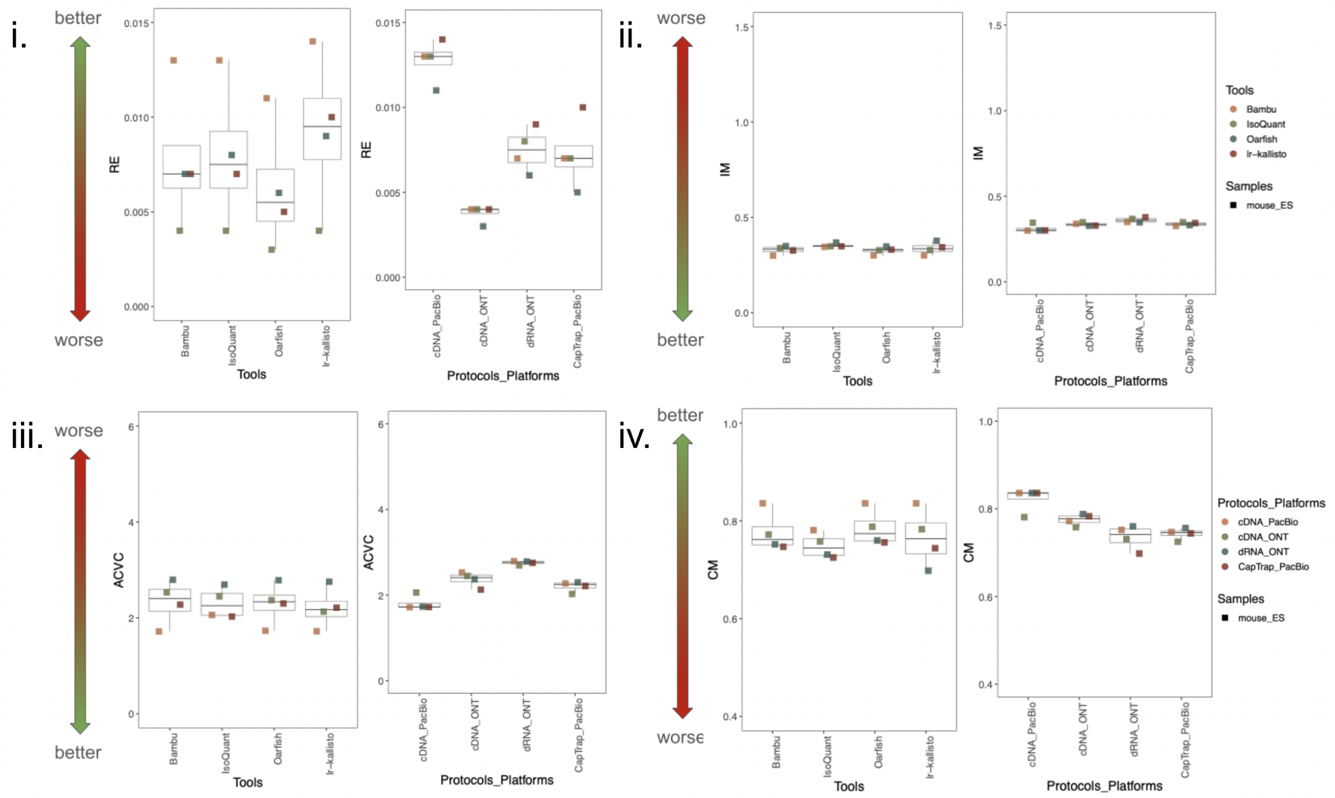

Fig D: Evaluation of lr-kallisto, Bambu, IsoQuant and Oarfish according to LRGASP challenge 2 metrics in Mouse ES cells.

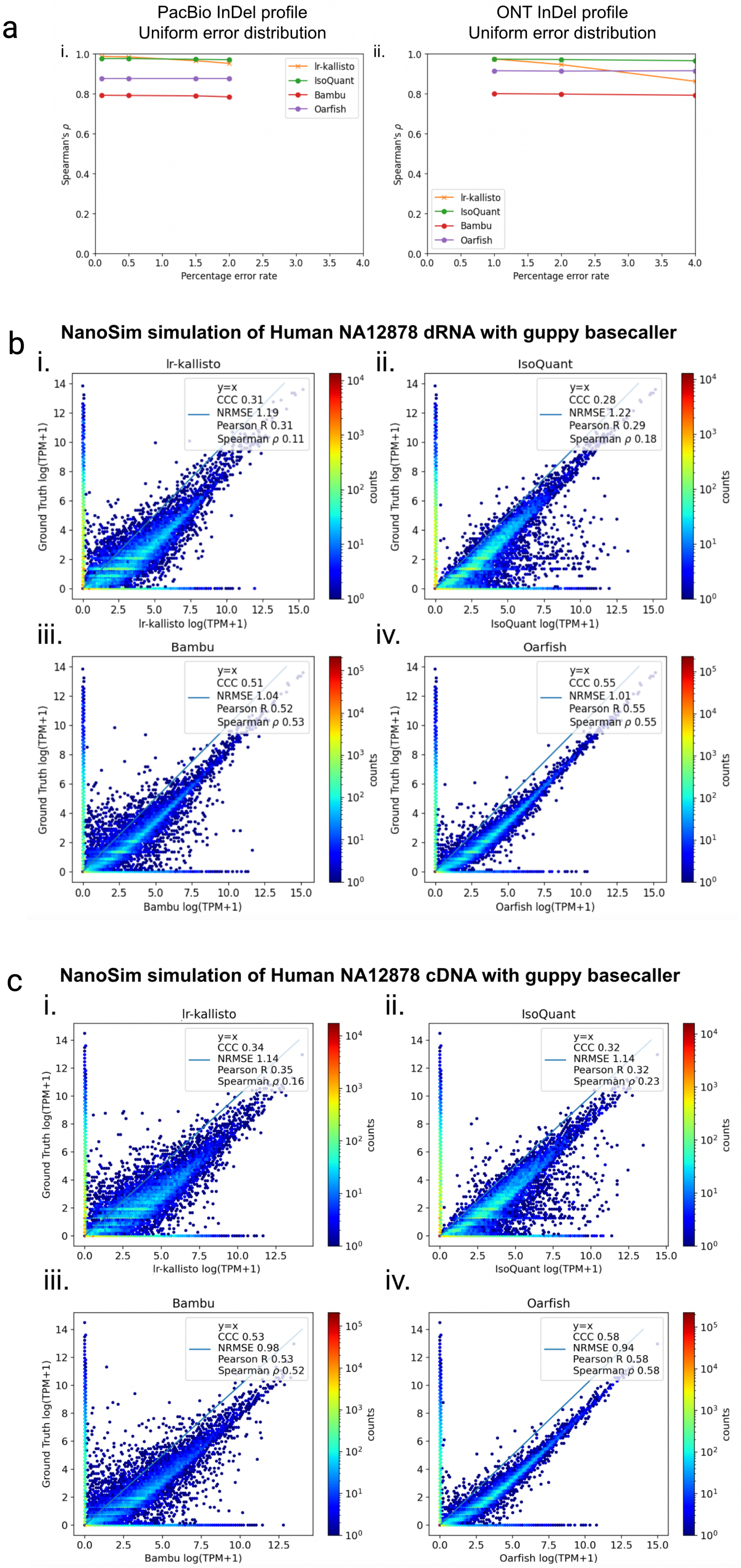

Fig E a) Benchmarks of Bambu, IsoQuant, lr-kallisto, and Oarfish on simulations with a range of error parameters. b) Performance on all annotated transcripts at ONT 11.2% sequencing error rate. c) Performance on all annotated transcripts at ONT 15.2% sequencing error rate.

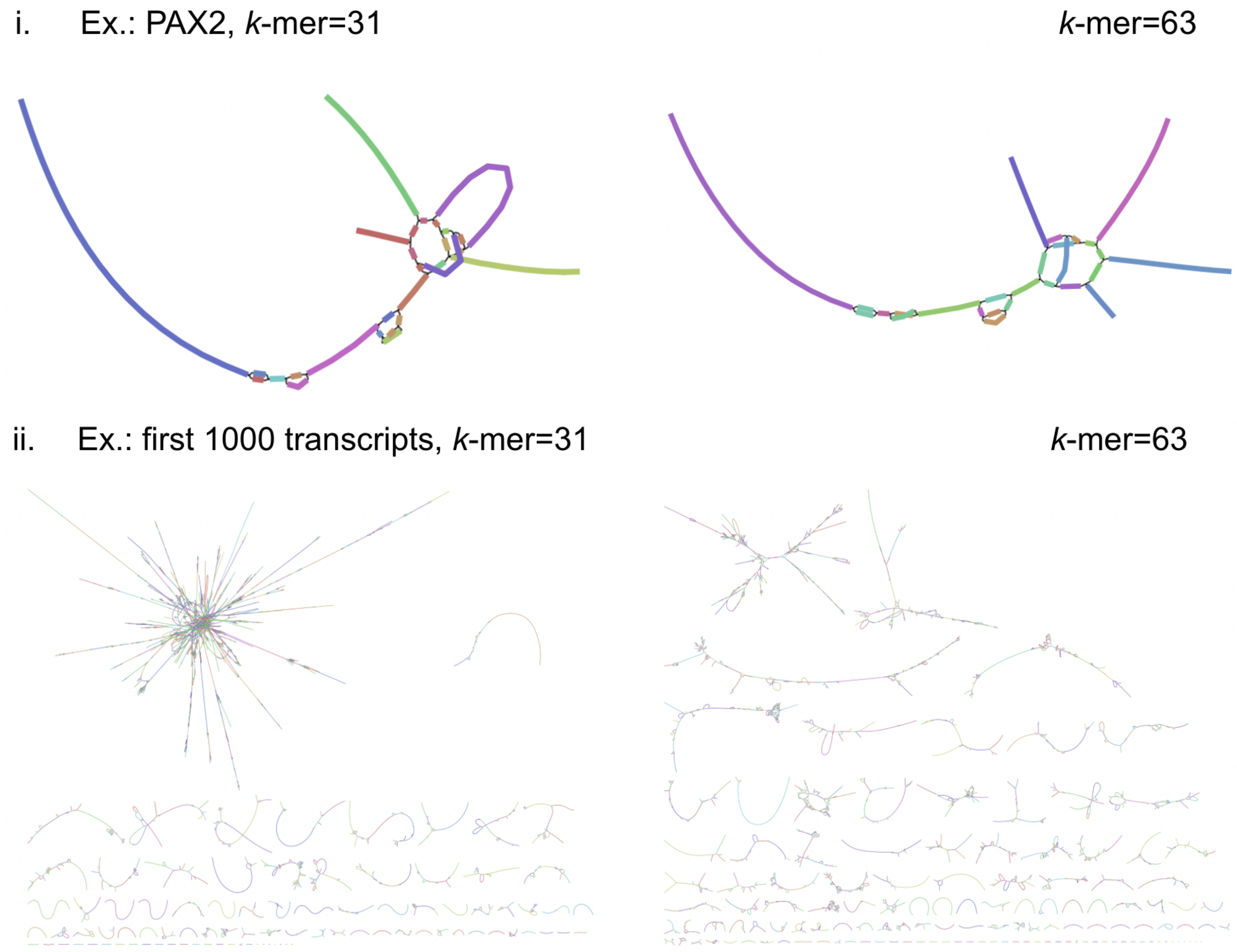

Fig F: lr-kallisto transcript de Bruijn Graph bandage plots.
