# Supplementary material for: Long-read sequencing transcriptome quantification with lr-kallisto
Source: PLoS Comput Biol. 2025 Dec 1;21(12):e1013692. doi: 10.1371/journal.pcbi.1013692 (PMC12680354; doi:10.1371/journal.pcbi.1013692)
Supplement: Attachment — Submitted filename: response_to_reviewers.pdf [file pcbi.1013692.s002.pdf]

Reviewers' comments:

Reviewer's Responses to Questions

Reviewer #1: **The paper by Loving et al proposed lr-kallisto, an updated version of Kallisto specific for long-read transcriptome quantification, which is relevant and useful in the context of fast growing interest in long read RNA sequencing technology. The authors evaluated the performance of lr-kallisto using multiple real datasets, and simulated datasets, and benchmarked lr-kallisto against other methods that are mostly used for long read quantification. In addition, the authors showed an application case of lr-kallisto on single-nuclei samples. In general, the manuscript is relatively clearly written with well presented results, except for some aspects that would need to be addressed or changed.**

See below for my comments in details:
Major comments:
1. Application on single-nuclei RNA-Seq samples is under-presented in several ways. Firstly, the conclusion in the first line of the paragraph (page 4 lines 69-70) mentions that efficient performance on single-cell/single-nuclei RNA-seq datasets, i.e., "is not only faster than other tools, but also benefits from the low-memory requirements of kallisto (23, 28)." is not well supported, citations 23, 28 are using kallisto, instead of lr-kallisto. Secondly, results presented in Supplementary Fig 2a, failed to highlight the role of lr-kallisto in this case, where gene level counts can be obtained easily with existing other methods, or even short read single-cell methods like Seurat. The authors should present how transcript level counts like, if possible, with comparison of other methods. Thirdly, the results on barcode seem distracting as it is mainly dependent on another method (splitcode), for better focus, the results on this can be shrinked.

Thanks for pointing out the need to specifically benchmark the memory usage and speed of lr-kallisto. While lr-kallisto and kallisto both benefit from the efficiency of pseudoalignment, we have now included direct benchmarks on lr-kallisto, finding that it retains the performance profile of kallisto, and in particular is much faster and more memory efficient than other tools which rely on genome alignments (see Supplementary Table 1). These benchmarks have been carefully performed to include benchmarking solely against the quantification time of other tools (i.e., not counting the time and memory they take to perform genome alignment). This was done at the request of another reviewer for an "apples-to-apples" comparison. We have also benchmarked the use of pseudobam with lr-kallisto (which provides genome alignment information), and in that case done head-to-head benchmarks with other tools.

In summary, even when the genome alignment step (typically with minimap2) is not included in the benchmark, lr-kallisto is much more efficient in comparison to other tools.

In regards to the second point, we have added Supplement Figure I Motivation, in which we compare kallisto to lr-kallisto on long-read data directly, and find a significant performance improvement with lr-kallisto, highlighting the fact that while long-read data can be processed with short-read tools, there is a major performance improvement that can be obtained with methods optimized for long reads. Furthermore, in a new analysis (Supplementary Figure 2c), we further illustrate that the results in our paper are not specific to the short-read tool kallisto: we find that replacement of kallisto with salmon does not change the conclusions since the results obtained with kallisto are highly correlated with those of salmon. Thanks for raising this point; we believe these additional comparison now make a stronger case for lr-kallisto. We have further added an analysis at the transcript-level in exome capture. We show that exome capture decreases the capture rate of lnc-RNAs while increasing the rate of protein coding RNAs.

Regarding the third point, we now emphasize (the new figure) Supplementary Figure 1f, which shows a direct comparison of quantification at the single-cell transcript-level, rather than focusing on barcode identification. We have left the barcode results in for clarity and completeness, as it shows the ability of lr-

kallisto to be used in a manner that is effective for single-cell RNA-seq, but the emphasis in the text is on quantification.

2. The additional value of exome capture is now under-demonstrated and confuses the focus of this paper. The main value of exome capture is probably more relevant to single-nuclei RNA-seq samples, which can be better presented to highlight beneficial impact of exome capture for single-nuclei samples, by showing the results as presented in Fig 1 b-c, Supplementary Fig. 1a for each nuclei, instead of bulk. Another thing is the analysis regarding random oglio vs 3' primer design needs more investigation. In particular, for page 4 lines 79 to 80 where between exome capture and non-exome capture, CCC is not as high as among exome capture ONT and Illumina, or non-exome capture ONT and Illumina, and then the authors concludes the distorting results suspected from a mix of 3'-end and randomly primed sequencing. This conclusion can be easily proved by repeating the analysis for 3' end only reads, and randomly primed reads.

We further explored the difference in 3'-end and randomly primed read sequencing by looking at the mean expression of 3'-end (polyT) reads in a cell in ONT vs Illumina, in randomly primed (randO) reads in a cell in ONT vs Illumina, and when reads from the two priming methods are merged for a cell. We found that randomly primed reads in a cell exhibit greater correlation to the corresponding randomly primed reads in Illumina in the highest mean expressed 100 transcripts in ONT, than with 3'-end reads in a cell (new figure Supplementary Fig. 1f). We also found that the expression of long non-coding RNAs is significantly different between the exome capture and non-exome capture as expected (numbers added to the text in discussion of Supplementary Figure 1f).

3. Page 4 line 65-66 "In addition to being more accurate than other methods, lr-kallisto is also more computationally efficient (Supplementary Fig. 1c)" The comparison is not fair in the sense where alignments in each other method by itself is useful and can be used for downstream analysis. I,.e., for lr-kallisto, minimap2 still needs to be processed separately. A more fair comparison would be excluding minimap2 timing from these methods as they can use alignments immediately. My another concern for this comparison is that mouse data is rather small, a benchmark on human dataset if possible would be very helpful to show how lr-kallisto scales up to big datasets.

This is a great point, as many users rely on alignment, rather than pseudoalignments, for downstream analysis. To facilitate the use of lr-kallisto for such applications, we have now implemented pseudobam, which is an approach to obtain BAM formatted alignment information for reads, and was previously implemented for kallisto, within lr-kallisto. Therefore, users of lr-kallisto do not require the separate mapping of reads with minimap2, even to produce alignments. We also have added a demonstration of the scaling up of lr-kallisto to the exome capture dataset in comparison to the other tools. We have manually verified that reads aligned with pseudobam match alignments with minimap2. This feature is now available via download from the kallisto Github repository.

4. The manuscript presentation can be improved significantly in terms of figure presentation for supplementary figures, and also method description for lr-kallisto.

Thanks for this suggestion. We have now reformatted supplementary figures after including the analysis updates in response to the reviews.

Minor comments:
Supplement Fig 1b. The number of points N is missing, and it seems at first glimpse that IsoQuant have much less points. It would help if authors can explain why is that so?

Note that these plots are hexbin plots, so the sum of the transcripts in the transcripts/bin add up to the same number, though the number of plotted hexbins may vary.

Page 4 lines 71-72 “100% of barcodes from the ONT reads that passed filtering were also found in Illumina sequenced reads” 100% of ONT barcodes found in illumina vs only half of Illumina barcodes found in ONT, why is so? Is it because of sequencing depth, if increasing ONT sequencing depth, would the results remain the same?

In response to this question we have re-examined our barcode identification approach in the ONT data, which is relying on a carefully constructed “seqspec” that defines precisely where barcodes are located. We are currently extracting barcodes with splitcode. While it is difficult to assess what would happen with much deeper ONT sequencing, as we do not have such data, we do expect that with increased depth (i.e. a comparable number of error corrected barcodes) we would achieve a near 100% overlap of barcodes.

Page 4 lines 73-74 “Increased UMI depth per nucleus yields higher Spearman correlations, indicating that with deeper sequencing depth, short and long read correlations will only improve” What is the range of sequencing depth corresponding to the increasing UMI depth? What each point represent here?

We have clarified our descriptions of plots and now describe exactly what each point is and what the metrics indicate regarding performance.

Supplementary figure 1e: for illumina reads, is it kallisto instead here? The current representation is a bit confusing.

We have fixed the typo ix in the plot title; it is indeed kallisto being used on the Illumina not lr-kallisto.

Page 4 lines 83-85, what is the estimation method for Illumina dataset? Based on the y-axis label, it seems kallisto Illumina results is used for comparison against lr-kallisto results. But for comparison against oarfish results, the method for Illumina is unclear. Please specify the method used here and explain further on the potential impact of method for Illumina data.

We have added clarifications, especially regarding short read processing with edits to Supplement Figure 2c, to include a comparison of long read tools compared to Illumina quantification with kallisto vs salmon. We note that lr-kallisto vs kallisto is higher in correlation than Oarfish vs salmon as well as lr-kallisto vs salmon is higher in correlation than Oarfish vs kallisto. Thus, we do not believe there is a bias creating inaccuracy in lr-kallisto quantifications compared to kallisto short read quantifications. Specifically, our results are robust to the short-read aligner used, and are conveying a strong correlation between quantification with long-reads using lr-kallisto and quantification with short reads obtained from libraries produced from the same samples.

For the pseudobulking of the Illumina Parse data, we used kallisto for quantification. Parse single-nucleus short read data are not analogous to standard single-end bulk data, as Parse uses a combination of polyA priming and random hexamer priming. Therefore, we pseudobulked the counts without length normalization, which is not appropriate for this priming chemistry, yielding comparable quantifications to the single-nucleus quantifications for alignment, counting, and disambiguating multimapping reads.

Page 4 lines 86 to 87, the comparison between direct cDNA and direct RNA against Illumina results differ between replicate 3 and replicate 4, where for replicate 4, direct cDNA concordants are better than direct

RNA, while for replicate 3, direct RNA concordants are better. Hence, the conclusion is not accurately described. Would recommend to rephrase.

We have rephrased this paragraph.

Suppl Fig 2c. The method for Illumina used here is unclear and might impact how the results can be interpreted.

We have added clarifications, especially regarding short read processing with edits to Supplement Figure 2c to include a comparison of long read tools compared to Illumina quantification with kallisto vs salmon. We note that lr-kallisto vs kallisto is higher in correlation than Oarfish vs salmon as well as lr-kallisto vs salmon is higher in correlation than Oarfish vs kallisto. Thus, we do not believe there is a bias creating inaccuracy in lr-kallisto quantifications compared to kallisto short read quantifications.

Page 11 line 281: “the length bias in sampling longer transcripts less times”is it the opposite instead?

Thanks for identifying this bad language; it has been fixed.

Page 12 line 313: “We use the first 1 million aligning reads to compute these effective, transcript-specific lengths”Why use 1st million aligning reads? Would it be biased? How representative is it? This needs to be evaluated.

The use of the first million unordered reads for learning this distribution is standard with kallisto since its inception. The kallisto paper showed that this is enough data to learn the distribution.

Reviewer #2: **Loving et al. present lr-kallisto, a modification of the kallisto pseudomapper tailored for long-read PacBio and Nanopore data. They compare lr-kallisto with other state-of-the-art quantification tools using multiple datasets, including simulated data. They demonstrate the tool's performance on single-nucleus LRS RNA-seq, both with and without exome capture. They claim that lr-kallisto outperforms other tools in quantification accuracy when compared with Illumina data. Additionally, lr-kallisto is faster.**
This study makes a significant contribution to the field of long-read RNA-seq quantification, where faster tools are necessary due to the increasing throughput of sequencing technologies. The manuscript shows that lr-kallisto performs comparably or better than other quantification tools. However, I would like to see further details on how well lr-kallisto quantifies "difficult cases"—specifically, when alternative isoforms are expressed simultaneously and when reads are incomplete. This is crucial because the primary advantage of long-read RNA-seq is its ability to detect alternative isoforms, and tools utilizing these data should address this capability explicitly.
Specific Comments:
1. The authors show that lr-kallisto exhibits a stronger correlation with Illumina data than other tools. However, I wonder if short-read quantification was performed using kallisto. If so, this might introduce bias due to the expected high concordance between kallisto-based methods. This potential bias should be acknowledged in the discussion.

As we have noted above, we have now added clarifications, especially regarding short read processing with edits to Supplement Figure 2c, to include a comparison of long read tools compared to Illumina quantification with kallisto vs salmon. We note that lr-kallisto vs kallisto is higher in correlation than Oarfish vs salmon as well as lr-kallisto vs salmon is higher in correlation than Oarfish vs kallisto. Thus, we do not believe there is a bias creating inaccuracy in lr-kallisto quantifications compared to kallisto short read quantifications. Specifically, our results are robust to the short-read aligner used, and are conveying a strong correlation between quantification with long-reads using lr-kallisto and quantification with short

reads obtained from libraries produced from the same samples.

2. As a pseudomapper, lr-kallisto should not only be compared with quantification tools but also with mappers such as minimap2 and uLTRA. While I understand that direct comparisons might be challenging because kallisto does not perform conventional mapping, certain analyses could still be informative. For example, a key distinction between minimap2 and uLTRA is that uLTRA improves the identification of small exons. How well does lr-kallisto handle this scenario, particularly when alternative isoforms are expressed? This could be evaluated using simulated data.

This is a great question. To address it we have now included benchmarks on the SIRV Set 4 dataset, which is a curated set of synthetic isoforms to evaluate tools and sequencing libraries and platforms' ability to capture the expression of complex isoforms, long isoforms, and lowly expressed isoforms. Thus, SIRV Set 4 is very well suited to answering the question of how lr-kallisto performs when alternative isoforms are present and specifically to answer the question of how it performs on complex isoform structures (alternative start and end sites, mono-exonic, alternative splice sites, etc.). Figure 2c-d which we have added shows a benchmark of lr-kallisto, IsoQuant, bambu, and Oarfish's performance on SIRV Set 4 in LRGASP's Human WTC11. We also added simulated data based on SIRV Set 4. We find that lr-kallisto performs well on this set of complex isoforms in both benchmark and simulation, which is described from line numbers 132-141.

3. A common issue in long-read RNA-seq is the presence of truncated reads, particularly at the 5' end, which complicates the quantification of transcripts with alternative transcription start sites (TSS). How does lr-kallisto perform in such cases? Can the algorithm accurately quantify isoform expression when alternative TSSs are present? Again, this could be tested using simulated data.

As noted above, we have now checked this using a new dataset as well as a simulation that were especially designed for this purpose, and have found that lr-kallisto performs well even when faced with data from complex isoform structures.

4. The parameters used for generating simulated data should be described in greater detail. Specifically, the authors should clarify how multiple isoforms of the same gene were simulated, whether expression values were kept constant or varied and whether the simulated data allowed for the assessment of minor alternative isoforms.

Thanks for this request; we have made an effort to document simulation parameters more carefully in our Github repository which contains code for reproducing all the results of the paper. We have also added a more precise discussion of simulation parameters in the Methods section under Data Simulations.

5. The authors suggest that lr-kallisto, despite being reference-based, could be used to identify novel transcripts by assembling reads that are not pseudomapped. This claim seems overstated and is not demonstrated in the manuscript. However, it would be interesting to assess how many reads are not used for quantification and how this compares to other tools. While some relevant data is present, a clear table summarizing these numbers would be beneficial. This table should ideally include the percentage of unmapped reads for each tool including results from Illumina-based kallisto for reference.

Thanks for the suggestion; we have added this to Supplementary Table 1.

6. The authors use data that includes spike-ins, which provide a ground-truth dataset, yet the manuscript does not present quantification results for these spike-ins. Including this analysis would strengthen the study.

We have now added such analysis; see the response to question 2 above.

7. The performance of pseudomappers relies heavily on the availability of a well-annotated transcriptome, which is not available for many species. Moreover, long-read RNA-seq has consistently revealed thousands of novel transcripts in nearly every experiment. Excluding unannotated transcripts from quantification inherently affects the performance of these tools. It would be valuable to see an analysis using partially annotated transcriptomes, possibly through simulation. While this might be beyond the current scope of the manuscript, the authors should discuss these considerations more thoroughly.

This is a great point, and we agree that long-reads have the benefit of making discovery possible, alongside quantification of existing transcripts. We have started to explore addition of an assembly dimension to lr-kallisto, specifically using reads that did not pseudoalign. While careful benchmarking of this approach is beyond the scope of the current paper, we have added a discussion of this possible extension to the discussion.

Additional Remarks:

• The description of the lr-kallisto algorithm is difficult to follow, especially for readers unfamiliar with kallisto. I strongly recommend adding a graphical figure to illustrate the algorithm.

We have added figure 4 and 5 in the Methods section to address this as well as clarified the description of the algorithm.

• The manuscript states that when the TCC intersection of a read is empty, the most frequently occurring TCC is selected. However, what happens in cases of ties? This should be clarified.

We have clarified the description of the algorithm.

• Line 302: Please clarify which ground truth is being referenced.

We have clarified the simulation ground truth is referenced.

• Line 363: Indicate the exact number of spike-in reads used in the analysis.

Reviewer #3: **This manuscript describes an update in the RNA-seq quantification software kallisto to enable quantifying transcriptomes from long-read sequencing data. The described algorithm seems to properly address the problem of performing fast and accurate pseudoalignments with long-read higher error rates. The benchmarking results show a very good agreement with Illumina data and a competitive performance with other existing tools, while providing the very fast execution times of a pseudoalignment-based approach. In summary, this is a useful addition to the kallisto pipeline and to the quantification toolkit for long-read sequencing data. I only have a few minor comments that I'm listing below.**

Minor comments:

* There are probably too many panels in figures 1 and 3, which makes axes labels in scatter plots too small to be read comfortably. In fact, the label "counts" on the right-axis of panels d and e of figure 1 is cropped. a solution would be to produce more figures with fewer panels.

We have split figures to improve readability.

* Something I do not understand from the color map representing the counts in the scatter plots of figures 1 and 3 is that, according to that color map, blue corresponds to lower counts and red to higher counts. however, blue appears associated with higher log2 TPM values and red to lower ones. Should not this be the other way around?

We have clarified the type of plots made in the text. They are hexbin plots, so each bin contains a count for the number of transcripts that fall into the bin. Hence, these plots show that there are fewer highly expressed transcripts than lowly expressed transcripts.

* In page 7, line 152, "comptible" -> "compatible"

Thank you for catching this.

* In page 12, after line 312, the displayed equation has no number and its notation is somewhat unclear, I guess 'l_{reads aligning to t}' refers to the length of the reads, but this is not explicitly mentioned and the summation has no index. The term in the denominator uses also quite a non-standard mathematical notation. I'd recommend the authors to use a more standard notation with single letters, describing them in the main text.

Thank you. We have corrected the notation.

* In page 12, it is unclear to me what it means that an option has "reduced performance in the wild"?

Reduced performance in the wild refers to reduced performance on real data vs simulation. We have rewritten this phrasing in the manuscript so that it is scientifically accurate and not colloquial.

Have the authors made all data and (if applicable) computational code underlying the findings in their manuscript fully available?

The PLOS Data policy requires authors to make all data and code underlying the findings described in their manuscript fully available without restriction, with rare exception (please refer to the Data Availability Statement in the manuscript PDF file). The data and code should be provided as part of the manuscript or its supporting information, or deposited to a public repository. For example, in addition to summary statistics, the data points behind means, medians and variance measures should be available. If there are restrictions on publicly sharing data or code —e.g. participant privacy or use of data from a third party—those must be specified.

Reviewer #1: Yes

Reviewer #2: Yes

Reviewer #3: Yes

PLOS authors have the option to publish the peer review history of their article (what does this mean?). If published, this will include your full peer review and any attached files.

If you choose “no”, your identity will remain anonymous but your review may still be made public.

Do you want your identity to be public for this peer review? For information about this choice, including consent withdrawal, please see our Privacy Policy.

Reviewer #1: No

Reviewer #2: No

Reviewer #3: No

[NOTE: If reviewer comments were submitted as an attachment file, they will be attached to this email and accessible via the submission site. Please log into your account, locate the manuscript record, and check for the action link "View Attachments". If this link does not appear, there are no attachment files.]

Figure resubmission:

While revising your submission, please upload your figure files to the Preflight Analysis and Conversion Engine (PACE) digital diagnostic tool, https://pacev2.apexcovantage.com/. PACE helps ensure that figures meet PLOS requirements. To use PACE, you must first register as a user. Registration is free. Then, login and navigate to the UPLOAD tab, where you will find detailed instructions on how to use the tool. If you encounter any issues or have any questions when using PACE, please email PLOS at figures@plos.org. Please note that Supporting Information files do not need this step. If there are other versions of figure files still present in your submission file inventory at resubmission, please replace them with the PACE-processed versions.

Reproducibility:

To enhance the reproducibility of your results, we recommend that authors of applicable studies deposit laboratory protocols in protocols.io, where a protocol can be assigned its own identifier (DOI) such that it can be cited independently in the future. Additionally, PLOS ONE offers an option to publish peer-reviewed clinical study protocols. Read more information on sharing protocols at https://plos.org/protocols?utm_medium=editorial-email&utm_source=authorletters&utm_campaign=protocols
