# Supplementary material for: Long-read sequencing transcriptome quantification with lr-kallisto
Source: PLoS Comput Biol. 2025 Dec 1;21(12):e1013692. doi: 10.1371/journal.pcbi.1013692 (PMC12680354; doi:10.1371/journal.pcbi.1013692)
Supplement: Attachment — Submitted filename: lr-kallisto_Response2_PlosCompBio.pdf [file pcbi.1013692.s003.pdf]

Reviewer #1: I appreciate authors' efforts in addressing my questions, most of them are addressed now with some minor points remained.
1. Supplementary Table 1: please explain what is d-list. Also, for pseudobam, more details need to be mentioned in main text. For now, this is only mentioned in Figure legend. How comparable it is with minimap2 bam files, also uLTRA as mentioned by another reviewer.

Thank you for asking for clarity on the d-list and the pseudobam. We have added the following text to explain the d-list and pseudobam:

- We also made lr-kallisto compatible with the d-list option of kallisto. The d-list option produces an index with distinguishing flanking k-mers that serve as certificates to ensure that reads are not mapped erroneously to exons when, in fact, they may have originated from intronic sequence.

- We include the pseudobam option in lr-kallisto, which outputs the pseudoalignments produced by kallisto in bam format, enabling users to analyze where particular reads have aligned.

The pseudobam option was introduced a while ago and comparisons with genome alignment were performed here: https://www.biorxiv.org/content/10.1101/444620v1 While pseudobam does not produce a base-by-base alignment, it does report for every read the transcriptomic / genomic coordinates of alignment. We have added this reference to the paper for clarity.

2. The barcode overlap between ONT and Illumina is still a bit confusing to me as I am unclear why would near 100% overlap is expected, given that less than half of the barcodes in Illumina can be found in ONT. Why would ONT barcodes all found in Illumina? I would suggest authors to either soften their speculation on this, or provide additional support for the current speculation.

The emphasis on our expectation of high overlap between ONT and Illumina is due to the experimental setup of sequencing, where a highly mixed sample with molecules from all cells is present in both the Illumina and ONT sequencing samples. In a thought experiment where the sequencing of the libraries was carried to totality, i.e. achieving full saturation of the molecules present in the library, we reckoned we would expect to achieve a 100% overlap. However you are correct that this estimation is based on an assumption that indeed the libraries are not fully sequenced, and that if they were they would totally agree. Estimating the extent of library saturation is complex, and instead we decided to test the saturation by downsampling. When we downsample the ONT to half of the present depth, we indeed recover only 85% of the barcodes found in the full ONT sample, suggesting that we are far from full saturation.

In the revision we have discussed this result now, and also toned down the speculation given the caveats as you suggested.
